# Triple-Layer Porous Transport Layers with Ultra-High Porosity for Enhanced Oxygen Transport and Catalyst Utilization in Water Electrolysis

**DOI:** 10.1007/s40820-025-01831-z

**Published:** 2025-06-30

**Authors:** Seong Hyun Park, Young Je Park, Seungsoo Jang, Pilyoung Lee, Soobin Yoon, Young-June Park, Chi-Young Jung, Kang Taek Lee

**Affiliations:** 1 Department of Mechanical Engineering, KAIST, Daejeon, 34141 Republic of Korea; 2 Hydrogen Research and Demonstration Center, Hydrogen Energy Institute, Korea Institute of Energy Research (KIER), Jeollabuk-do, 56332 Republic of Korea; 3 Hydrogen and Fuel Cell Development Center, Hyundai Motor Group, Gyeonggi-do, 16891 Republic of Korea; 4 KAIST Graduate School of Green Growth & Sustainability, Daejeon, 34141 Republic of Korea

**Keywords:** Proton exchange membrane water electrolysis, Porous transport layer, Catalyst utilization, Mass transport, Digital twin

## Abstract

The commercialization of proton exchange membrane water electrolysis (PEMWE) for green hydrogen production hinges on the development of low-cost, high-performance titanium porous transport layers (PTLs). This study introduces a triple-layer Ti-PTL with a graded porous structure and a 75% ultra-high porosity backing layer, fabricated through tape casting and roll calendering. This triple-layer PTL, composed of a microporous layer, an interlayer, and a highly porous backing layer, enhances catalyst utilization, mechanical integrity, and mass transport. Digital twin technology using X-ray revealed increased contact area and triple-phase boundary at the interface with the catalyst layer, significantly improving oxygen evolution reaction kinetics. Numerical simulations demonstrated that the strategically designed porous structure of the triple-layer PTL facilitates efficient oxygen transport, mitigates oxygen accumulation, and improves reactant accessibility. Electrochemical evaluations showed improved performance, achieving 127 mV reduction in voltage at 2 A cm^−2^ compared to a commercial PTL, highlighting its potential to enhance PEMWE efficiency and cost-effectiveness.

**Supplementary Information:**

The online version contains supplementary material available at 10.1007/s40820-025-01831-z.

## Highlights

- A novel triple-layer Ti-porous transport layer (PTL), fabricated using a practical and scalable tape casting and roll calendering process, enhances catalyst utilization by increasing interfacial contact area and the triple-phase boundary.
- The ultra-high porosity (75%) backing layer and graded structure maximize oxygen transport, mitigate oxygen accumulation, and improve reactant accessibility.
- Electrochemical evaluations demonstrate a 127 mV reduction in voltage at 2 A cm^−2^ compared to a commercial PTL, accelerating proton exchange membrane water electrolysis commercialization and supporting the transition to sustainable energy.

**Supplementary Information:**

The online version contains supplementary material available at 10.1007/s40820-025-01831-z.

## Graphical abstract

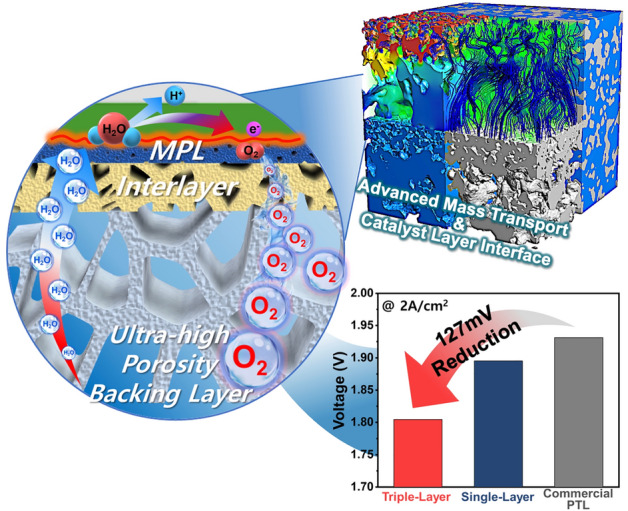

## Introduction

The increasing global demand for energy has intensified the urgency to develop eco-friendly energy solutions to combat climate change [1–3]. Transitioning from fossil fuel-based systems to sustainable energy sources is critical in achieving a carbon–neutral future. Among these sources, hydrogen energy has gained significant attention for its potential as a clean energy and sustainable alternative [4–6]. In particular, green hydrogen, produced via water electrolysis powered by renewable energy sources such as solar and wind, has emerged as a key pillar of this transition. Distinguished by its zero greenhouse gas emissions, green hydrogen offers an environmentally friendly pathway compared to traditional hydrogen production methods reliant on fossil fuels. Proton exchange membrane water electrolysis (PEMWE) is one of the most promising technologies for producing green hydrogen. This technology utilizes a polymer electrolyte membrane to split water into hydrogen and oxygen, offering benefits such as high current density, rapid system response, and low operating temperatures [7–9]. These attributes position PEMWE as a mature and reliable hydrogen production technology.

Despite these advantages, challenges remain in achieving the widespread commercialization of PEMWE systems, with high stack cost being a primary obstacle [10, 11]. The stack comprises costly components, including the catalyst-coated membrane (CCM), bipolar plate (BPP), and porous transport layer (PTL), significantly contributing to the overall system cost [12, 13]. Addressing this issue requires innovative approaches, such as reducing material usage, developing cost-effective manufacturing processes, or improving system performance. Enhancing cell performance, in particular, can increase hydrogen production efficiency, ultimately lowering the cost per unit of hydrogen [14, 15]. Among these components, the PTL accounts for a substantial share of the PEMWE stack cost due to the requirement for corrosion-resistant Ti materials at the anode [16–18]. In particular, the manufacturing process of Ti materials with complex pore structures that facilitate efficient oxygen and water transport contributes substantially to the overall PTL cost. Furthermore, the microstructural characteristics of the PTL play a critical role in the performance of PEMWE cells. Its surface structure influences catalyst utilization at the anode catalyst layer interface, while pore structure attributes such as pore size and porosity affect mass transport efficiency [19, 20].

Recent research efforts have focused on developing advanced manufacturing techniques and optimizing PTL performance. For instance, Mo et al*.* introduced a wet etching method to fabricate PTLs with tunable straight pores at reduced costs [21]. Other studies have sought to balance mass transport and interfacial contact by optimizing PTL porosity, with sintered powder PTLs showing optimal performance at 30–40% porosity [22, 23]. To overcome the trade-off between mass transport and interfacial contact with the catalyst layer, multilayer PTLs incorporating microporous layers (MPLs) have also been explored [24–26]. Lettenmeier et al*.* showed performance improvements in PEMWEs by vacuum plasma spraying MPLs onto commercial PTLs [27]. Additionally, Schuler et al*.* achieved up to a 100 mV voltage reduction at a current density of 2 A cm^−2^ compared to commercial PTLs by stacking MPLs with different particle sizes [28]. Stiber et al*.* demonstrated that a hierarchical porous structure composed of a Ti sintered layer deposited onto a Ti mesh effectively facilitated two-phase transport, resulting in a cell voltage of approximately 2.2 V at a high current density of 4 A cm^−2^ [29].

**Fig. 1:**
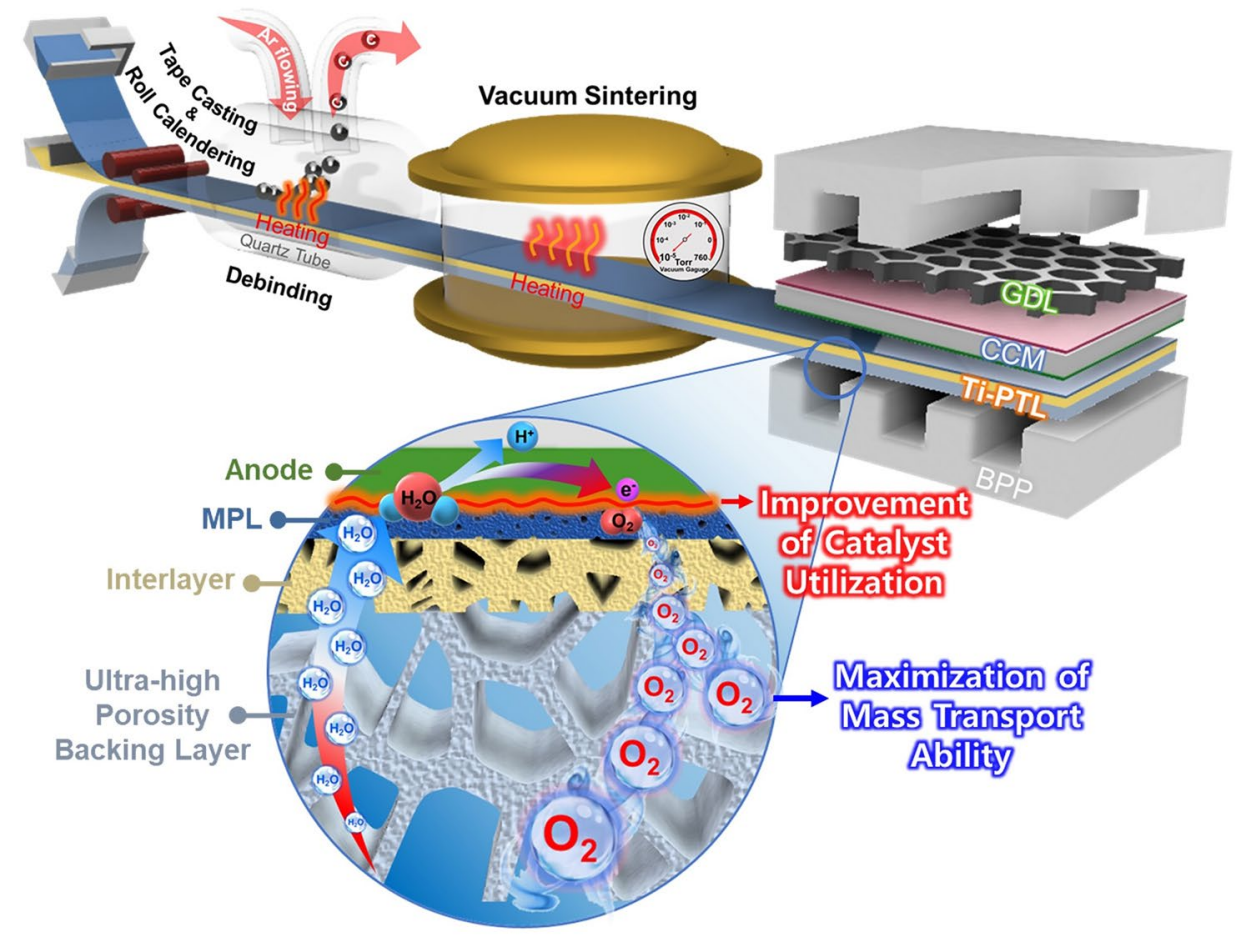
Schematic representation of a triple-layer PTL manufactured using tape casting process combined with the lamination-calendering procedure

Despite considerable efforts, a pressing need for the development of optimal PTL structures with practical fabrication processes to achieve high performance remains unmet. The fabrication strategies proposed in previous studies often lack the precision required to construct PTLs with well-controlled multilayered pore architectures. Furthermore, there remains a limited understanding of the mechanisms governing catalyst layer-MPL interfacial enhancement and two-phase mass transport in the graded PTL structures. To address these challenges, tape casting offers a highly effective solution. This straightforward fabrication technique is well-suited for large-area production and mass production, providing superior cost-effectiveness and fine control over pore structure compared to conventional PTL fabrication methods [30, 31]. In addition, to achieve high-performance Ti-PTLs, we implemented a novel triple-layer structure with a medium-porosity interlayer positioned between the MPL and a highly porous backing layer, as illustrated in Fig. 1. The design aims to improve catalyst utilization by ensuring the robust contact interface between the catalyst layer and MPL, while maximizing mass transport through the graded porous structure. Moreover, the interlayer enhances mechanical flexibility by integrating layers with highly contrasting pore sizes and porosities.

In this study, we present a heterogeneous pore-graded triple-layer PTL fabricated via a scalable tape casting and lamination-calendering process. Advanced 3D microstructural visualization and quantification, achieved through digital twin technology using X-ray microscopy (XRM), were employed to analyze the interfacial properties of the PTL and catalyst layer, demonstrating superior catalyst utilization. Furthermore, numerical simulation provided an in-depth analysis of mass transport enhancements, and the electrochemical performance of PEMWE cells incorporating the triple-layer PTL was evaluated to establish its feasibility for practical water electrolysis applications.

## Experimental Section

### Fabrication of Ti-PTLs

Ti-powders (Sejong Materials) were mixed with a carefully adjusted composition of ethanol (Sigma Aldrich), di-n-butyl phthalate (DBP, Junsei Chemical), polyvinyl butyral (PVB, Eastman Chemical Company), and poly(methyl methacrylate) (PMMA, ASP Corp.) to prepare a slurry tailored to each layer in the tape casting process. The Ti-slurry was poured into the doctor blade for tape casting. A roll laminator (EXCELAMII-355Q, GMP) was used to stack the resulting green tapes for each layer at 110 °C. The stacked green tapes were placed in a quartz tube furnace and a debinding process was performed in ultra-high purity Ar atmosphere. After debinding, the laminated tapes were sintered in a vacuum furnace under a pressure of 10^–5^ Torr. Finally, a platinum coating was applied to the sintered Ti-PTL to prevent the formation of an oxide layer.

### Characterization

To examine the microstructure of the Ti-PTLs and the morphology of the Ti-powders, scanning electron microscopy (SEM, JSM-IT800, JEOL) was used. Thermogravimetric analysis (TGA, TG209F1Libra, Netzsch) was conducted to determine the debinding temperature profile, with measurement conducted from room temperature to 800 °C at a heating rate of 5 °C min^−1^ in the Ar atmosphere. Phase-purity of titanium was verified via X-ray diffraction (XRD, SmartLab, Rigaku) over a 2θ range of 20°–80° using Cu Kα radiation (λ = 1.5418 Å). The mechanical property of the PTL was assessed through a compression test (Z050, Zwick/Roell) under a constant loading rate of 1 kN min^−1^﻿. To measure the contact resistance of the PTL, the voltage response to an applied current was measured across an annular PTL sample like a doughnut shape. The measurement was carried out with reference to the contact resistance values at the points of contact between the upper and lower jigs. The contact resistance was calculated considering thickness and cross-sectional area of PTLs, as shown in Eq. (1) [32].

1 $$\rho = \frac{V}{I}\times \frac{A}{h}$$

where $\rho$ is the resistivity, $V$ is the measured voltage, and $I$ is the applied current. $A$ and $h$ are the cross-sectional area and the PTL thickness. Surface roughness and morphology of the PTLs examined using 3D laser confocal microscopy (VK-X 3000, Keyence) at a pixel resolution of 277 nm.

### Digital Twin Using X-Ray Microscope

An XRM (Xradia 520Versa, Zeiss) was used to analyze the microstructure of the Ti-PTL and the contact interface between the catalyst layer and PTL. For PTL microstructure analysis, X-ray scanning was performed at 150 kV acceleration voltage, 20 × magnification, and a 10 s exposure time, achieving a voxel size of 0.7 μm in the X, Y, and Z axis. To examine the contact interface between the catalyst layer and PTL in the assembled cell, X-ray scanning was conducted at acceleration voltages of 160 kV, with a magnification of 20 × and an exposure time of 6 s, resulting in a voxel size of 1 μm in all axes. 3D reconstruction of the acquired images was processed using AVIZO software, enabling the quantitative analysis of porosity, pore size, tortuosity, contact area, and triple-phase boundary (TPB).

### Numerical Simulation

Computational fluid dynamics simulations were conducted using the GeoDict software to analyze velocity streamlines and oxygen transport pressure distribution within the PTLs. The 3D reconstructed PTL structures, obtained from XRM tomography, were used as input for simulations. The computational domain was modeled as fully saturated with water, reflecting high current density operations. Pore-morphology-based numerical simulations were conducted using an incompressible Stokes equation derived from Darcy’s law:

2 $$\overrightarrow{u}=-\frac{k}{\mu }\left(\nabla P-\overrightarrow{f}\right)$$

3 $$-\mu \Delta \overrightarrow{u}+\nabla P=\overrightarrow{f}$$

where $\overrightarrow{u}$*, *$k$*, *$\mu$*,* $P$, and $\overrightarrow{f}$ are the fluid flow velocity, permeability, fluid viscosity, pressure and force density, respectively. Pressure drop within the PTL microstructures was calculated using Darcy’s law along with the Young–Laplace equation:

4 $$P_{c} = \frac{2\sigma \cos \cos \theta }{r}$$

where ${P}_{c}$ is capillary pressure, $r$ is the capillary radius, $\sigma$ is surface tension and $\theta$ is the contact angle. The contact angle in PTLs was similarly controlled to 46°. The internal and external boundary conditions of computational domain were applied as no-slip and symmetry conditions, respectively. A 1D time-dependent numerical simulation was introduced using the COMSOL software to analyze the saturation of oxygen transport within the PTL structure. The model is grounded in the mass conservation equation for two-phase transport in porous media, expressed as follows:

5 $$\frac{\partial }{\partial t}\left( { \in s_{i} \rho_{i} } \right) + \nabla \cdot \left( {\rho_{i} \vec{u}_{i} } \right) = S_{m}$$

where $\epsilon$ is porosity, $s$ is the volume fraction, $\rho$ is the density, and ${S}_{m}$ is the source term. Here, the subscript $i$ represents species $i$, where $i=1$ and $i=2$ correspond to water and oxygen, respectively. The momentum conservation equation is derived from Darcy’s law:

6 $$\vec{u}_{i} = - \frac{{\kappa_{0} \kappa_{r,i} }}{{\mu_{i} }}\nabla p_{i}$$

where $\kappa_{0}$ is the absolute permeability and $\kappa_{r}$ is the relative permeability. The absolute permeability is calculated using the Kozeny-Carman model:

7 $$\kappa = \frac{{d_{p}^{3} }}{180}\frac{{ \in^{3} }}{{\left( {1 - \smallint } \right)^{2} }}$$

where $d_{p}$ is the particle diameter. To enhance the model’s through-plane interpretation, both particle diameter and porosity were specified as distributed values with 1 μm intervals along the thickness direction extracted from the tomography-based reconstructed 3D structure. The relative permeability depends on the phase saturation and is expressed as:

8 $$\left\{ {\begin{array}{*{20}l} {\kappa_{r,1} = s_{1}^{3} } \hfill \\ {\kappa_{r,2} = \left( {1 - s_{1} } \right)^{3} = s_{2}^{3} } \hfill \\ \end{array} } \right.$$

The capillary pressure is defined as the pressure difference between the two species and can be calculated as:

9 $$p_{ca} = p_{2} - p_{1} = \sigma \cos \cos \left( \theta \right) \left( {\frac{ \in }{{\kappa_{0} }}} \right)^{0.5} J\left( {s_{1} } \right)$$

Here, the Leverett-J function, $J\left( {s_{1} } \right)$, is used to represent the correlation between capillary pressure and water saturation in PTL:

10 $$J\left( {s_{1} } \right) = \left\{ {\begin{array}{*{20}l} {1.42\left( {1 - s_{1} } \right) - 2.12\left( {1 - s_{1} } \right)^{2} + 1.26\left( {1 - s_{1} } \right)^{3} , \theta < 90^\circ } \hfill \\ {1.42s_{1} - 2.12s_{1}^{2} + 1.26s_{1}^{3} , \theta > 90^\circ } \hfill \\ \end{array} } \right.$$

### Electrochemical Analysis

A CCM with a Nafion 115 membrane composed of IrO₂ (0.5 mg cm^−2^) as the anode catalyst and Pt/C (0.5 mg cm^−2^) as the cathode catalyst was used. The fabricated Ti-PTL was loaded on the anode side, while carbon paper was used on the cathode side. A PEMWE cell with an active area of 5 cm^2^ was assembled under a compressive force of 12.4 Nm torque. For electrochemical measurement, deionized water at 80 °C was supplied to the cell at 15 mL min^−1^, with cell temperature maintained at 80 °C during testing using an in-house test bench. For the voltage breakdown analysis, a potentiostat (VMP-300, Bio-Logic) was employed to obtain high-frequency resistance (HFR) and Tafel plots. The cell voltage ($E_{{{\text{cell}}}}$) can be expressed as the sum of the reversible cell potential ($E_{{{\text{rev}}}}^{0}$) and three overpotentials ($\eta_{{{\text{ohm}}}}$, $\eta_{{{\text{kin}}}}$, $\eta_{{{\text{mtx}}}}$) as follows:

11 $$E_{{{\text{cell}}}} = E_{{{\text{rev}}}}^{0} + \eta_{{{\text{ohm}}}} + \eta_{{{\text{kin}}}} + \eta_{{{\text{mtx}}}}$$

where $\eta_{{{\text{ohm}}}}$, $\eta_{{{\text{kin}}}}$, and $\eta_{{{\text{mtx}}}}$ represent the ohmic, kinetic, and mass transport overpotentials, respectively. The $\eta_{{{\text{ohm}}}}$, determined as the product of HFR and current density ($i$), was calculated as:

12 $$\eta_{{{\text{ohm}}}} = i \times HFR$$

The $E_{{iR - {\text{free}}}}$ was calculated by subtracting $\eta_{{{\text{ohm}}}}$ from the $E_{{{\text{cell}}}}$:

13 $$E_{{iR - {\text{free}}}} = E_{{{\text{cell}}}} - \eta_{{{\text{ohm}}}}$$

The $\eta_{kin}$ is defined using the Tafel model, as follows:

14 $$\eta_{{{\text{kin}}}} = b \times \log \frac{i}{{i_{0} }}$$

where $b$ is the Tafel slope at low current density (0.01–0.1 A cm^−2^), and $i_{0}$ is the exchange current density. The $\eta_{{{\text{mtx}}}}$ was calculated by subtracting the $E_{{{\text{rev}}}}^{0}$, the $\eta_{{{\text{ohm}}}}$, and the $\eta_{{{\text{kin}}}}$ from the $E_{{{\text{cell}}}}$:

15 $$\eta_{{{\text{mtx}}}} = E_{{{\text{cell}}}} - E_{{{\text{rev}}}}^{0} - \eta_{{{\text{ohm}}}} - \eta_{{{\text{kin}}}}$$

## Results and Discussion

### Fabrication of Triple-Layer PTL with an Ultra-High Porosity Backing Layer

**Fig. 2:**
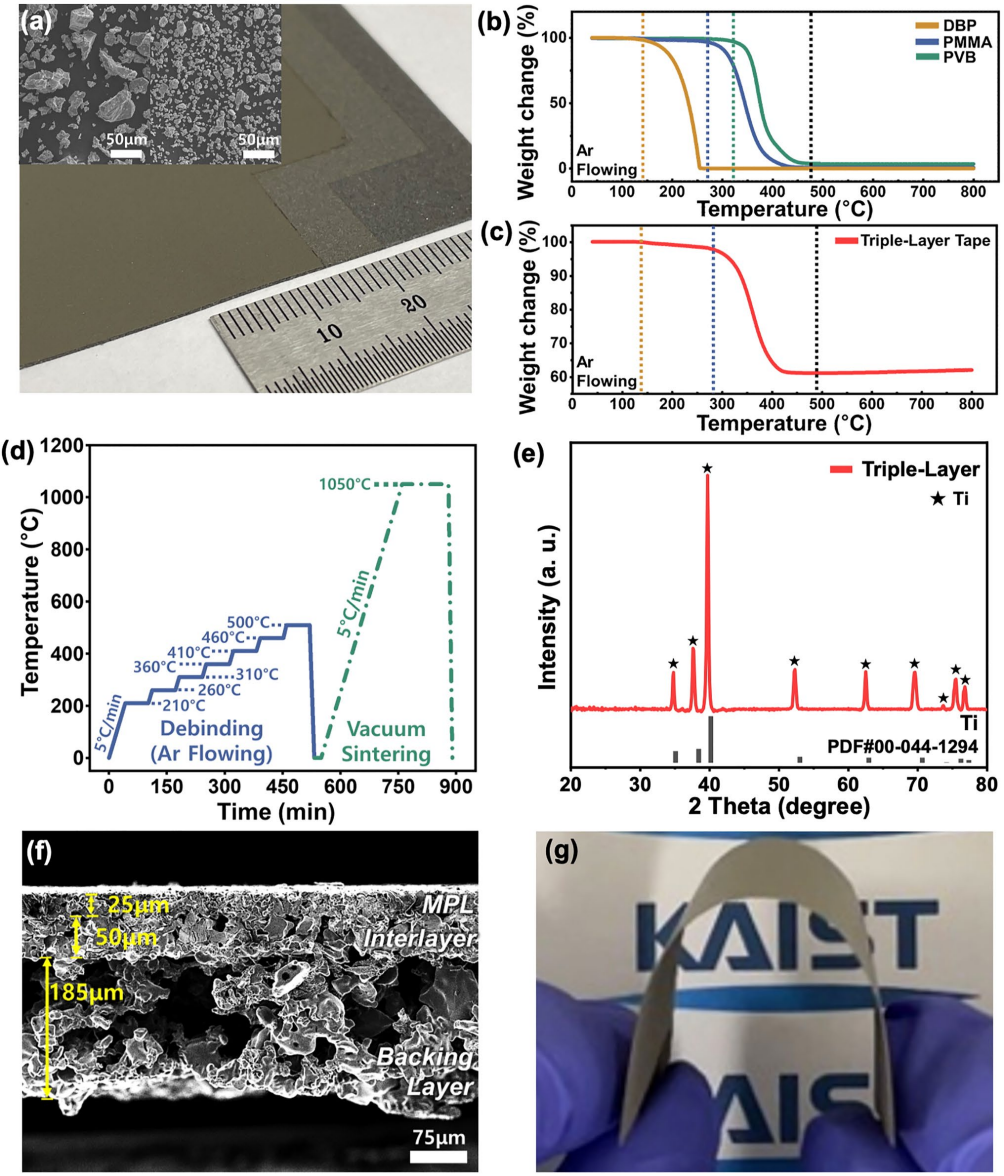
Fabrication process for a triple-layer PTL with an ultra-high porosity backing layer. **a** Ti-green tape with a triple-layer structure comprising MPL, interlayer, and backing layer, stacked through a roll calendering process, and SEM images of Ti-powder for MPL (left) and interlayer (right) and backing layer (right). TGA curves of **b** DBP, PMMA, and PVB, and **c** triple-layer tape. **d** Temperature profile for the debinding process and vacuum sintering process. **e** XRD pattern of triple-layer PTL. **f** Cross-sectional SEM image of triple-layer PTL. **g** Photograph of a triple-layer PTL with an ultra-high porosity backing layer showing superior mechanical flexibility

Figure 2a illustrates the green tape of a triple-layer structure, fabricated by stacking three distinct layers using a roll calendering process. The MPL green tape was prepared through tape casting using a slurry containing fine Ti-powder (d₅₀ = 13.6 μm), which was intentionally selected to induce a uniform and low-roughness surface. In contrast, the interlayer and backing layer green tapes were manufactured using slurries with large Ti-powder (d₅₀ = 38.4 μm) (inset of Fig. 2a). For the backing layer slurry, PMMA was added to achieve a highly porous structure. To ensure high electrical conductivity and mechanical stability, the heat treatment process was meticulously engineered to avoid the formation of undesirable insulating phases, such as TiC or TiO_x_. Organic additives like binders, plasticizers, and PMMA in the Ti-green tape pose a significant risk of forming thermodynamically stable Ti-C bonds, which result in TiC. Therefore, a precise debinding procedure was implemented to completely remove these organic components prior to sintering. TGA was conducted on the organic additives and triple-layer green tape under an Ar atmosphere to develop the debinding procedure. The TGA results indicated that decomposition of organic additives (DBP, PMMA, and PVB) began at − 140 °C and was complete by − 480 °C (Fig. 2b). Similarly, TGA result of the triple-layer green tape confirmed that significant weight loss occurred within the same temperature range, indicating the effective removal of organic materials (Fig. 2c). However, weight gain observed above 500 °C (Fig. S1) suggested the onset of Ti oxidation, even in the Ar atmosphere. Based on these observations, an optimized temperature profile was established to achieve phase-pure Ti-PTL with high electrical conductivity and mechanical integrity as illustrated in Fig. 2d. The XRD pattern confirms the successful fabrication of phase-pure triple-layer Ti-PTL (Fig. 2e). Figure 2f shows a cross-sectional SEM image of the resulting triple-layer Ti-PTL, revealing a robust and well-integrated Ti network with no evidence of delamination. The graded porous structure, consisting of a thin MPL (25 μm) and a highly porous backing layer, was distinctly visible. Surface SEM images of the MPL and backing layer demonstrated a stark contrast in porosity and pore size between the two layers reflecting their functional distinctions (Fig. S2). As a reference, a single-layer PTL with 30–40% porosity was additionally fabricated (Fig. S3). In contrast, a dual-layer Ti-PTL configuration, consisting of an MPL directly stacked on a backing layer without an interlayer, suffered from poor mechanical stability following heat treatment. This instability resulted in delamination and fragmentation due to the extreme differences in pore structure and the ultra-high porosity of the backing layer (Fig. S4). Remarkably, the triple-layer PTL demonstrated excellent mechanical flexibility and freestanding capability (Fig. 2g), even with the high porous backing layer. In addition, compression testing revealed that the structure remained stable under an assembly pressure of 150 N cm^−2^, which corresponds to the typical compression pressure applied in PEMWE, and exhibited sufficient compressive strength to withstand the electrolysis differential pressure of 50 bar (Fig. S5) [29, 33, 34]. This superior mechanical property is attributed to the inclusion of the interlayer, which effectively integrates the contrasting pore structures of the MPL and the backing layer, ensuring mechanical robustness.

### 3D Pore Structure Analysis of Ti-PTLs

**Fig. 3:**
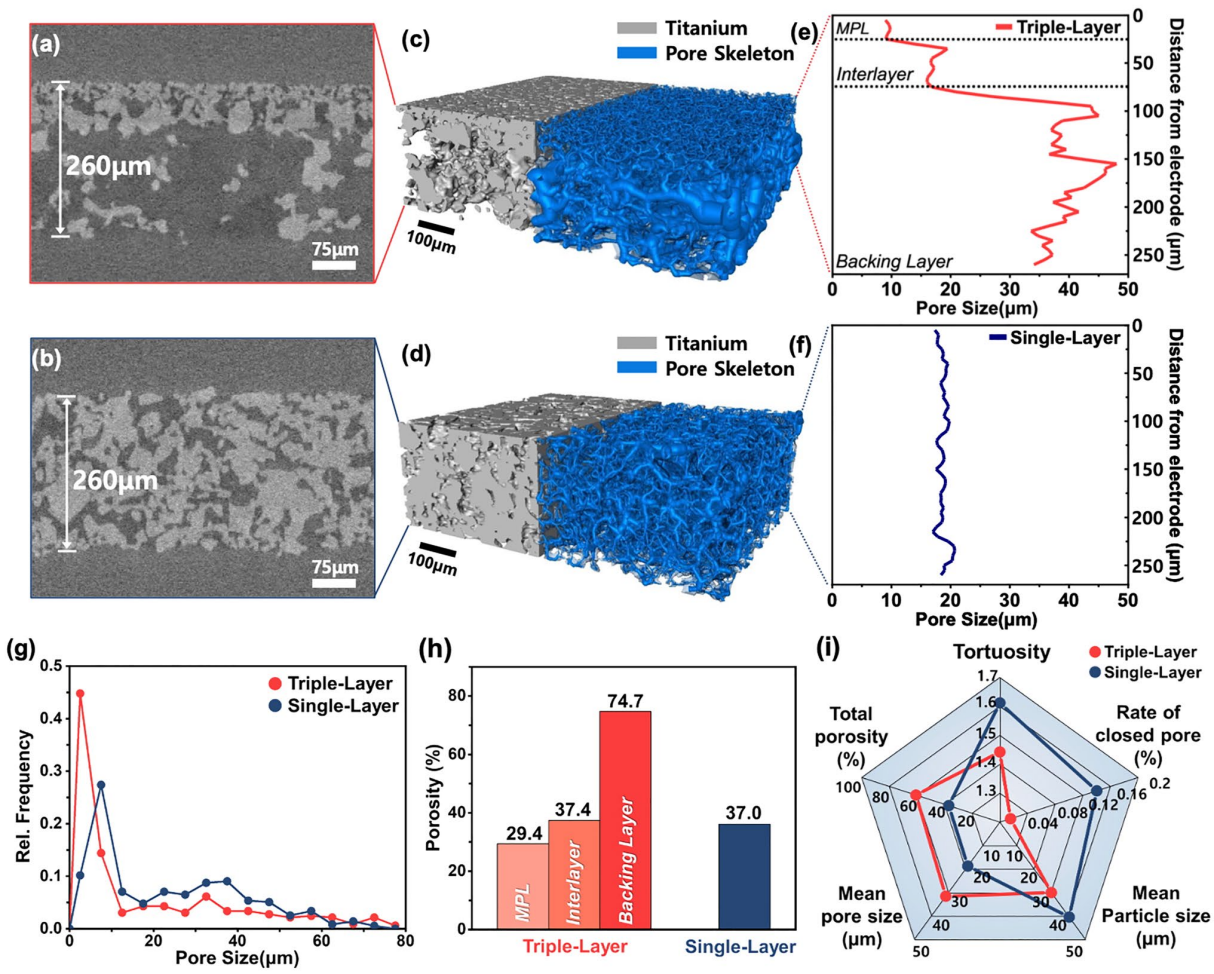
Pore-structural characteristics of triple-layer PTL and single-layer PTL. Cross-sectional XRM images of **a** triple-layer PTL and **b** single-layer PTL. Reconstructed 3D microstructures and pore skeletons of **c** triple-layer PTL and **d** single-layer PTL. Local pore size as a function of distance from the electrode for **e** triple-layer PTL and **f** single-layer PTL. **g** Pore size distributions across the entire structure of each PTL. **h** Comparison of porosity. **i** Radar chart for comparative analysis of microstructural properties

To perform an in-depth microstructural analysis, a state-of-the-art 3D reconstruction process was conducted, utilizing digital twin technology via XRM (Fig. S6). Figure 3a, b presents cross-sectional XRM images of the triple-layer PTL and single-layer PTL, respectively. Although both PTLs have the same thickness of 260 μm, the triple-layer PTL exhibits a significantly more porous backing layer compared to the single-layer PTL. Figure 3c, d shows the reconstructed 3D microstructures and pore skeletons of the Ti-PTLs, visually highlighting the hierarchical pore structure in the triple-layer PTL. In contrast, the single-layer PTL displays a more uniform pore distribution throughout its structure. The quantitative analysis of these microstructures is summarized in Table S1. Figure 3e illustrates the local pore size distribution across the through-plane of the triple-layer PTL, affirming its distinct gradient pore structure. The observed pore size transitions align well with the designed layer thicknesses: the MPL (25 µm), interlayer (50 µm), and backing layer (185 µm), as previously shown in the cross-sectional SEM image (Fig. 2f). Conversely, the single-layer PTL demonstrates a uniform pore structure across its thickness, with a mean pore size of 18.5 μm (Fig. 3f). Figure 3g presents the pore size distributions across the entire structures of the triple-layer PTL and the single-layer PTL. Due to the formation of a large number of micropores within the MPL structure, the triple-layer PTL demonstrates a significantly higher proportion of micropores across the entire structure compared to the single-layer PTL. The porosity is further quantified in Fig. 3h, where the triple-layer PTL demonstrates a graded porosity profile. Particularly, the backing layer achieves an ultra-high porosity of 75%, which is considered exceptionally high for a sintered powder PTL (Table S2), indicating that the addition of an interlayer effectively facilitates this structure while maintaining mechanical integrity. In contrast, the single-layer PTL exhibits a consistent porosity of 37%, a range typically reported to optimize performance in single-layer powder-sintered PTLs [11, 22, 23]. Finally, a comparative analysis of key microstructural parameters is provided in Fig. 3i using a radar chart. The triple-layer PTL exhibits superior microstructural characteristics for mass transport efficiency, including a lower rate of closed pore and reduced tortuosity.

### Interfacial Contact Properties with Catalyst Layer

**Fig. 4:**
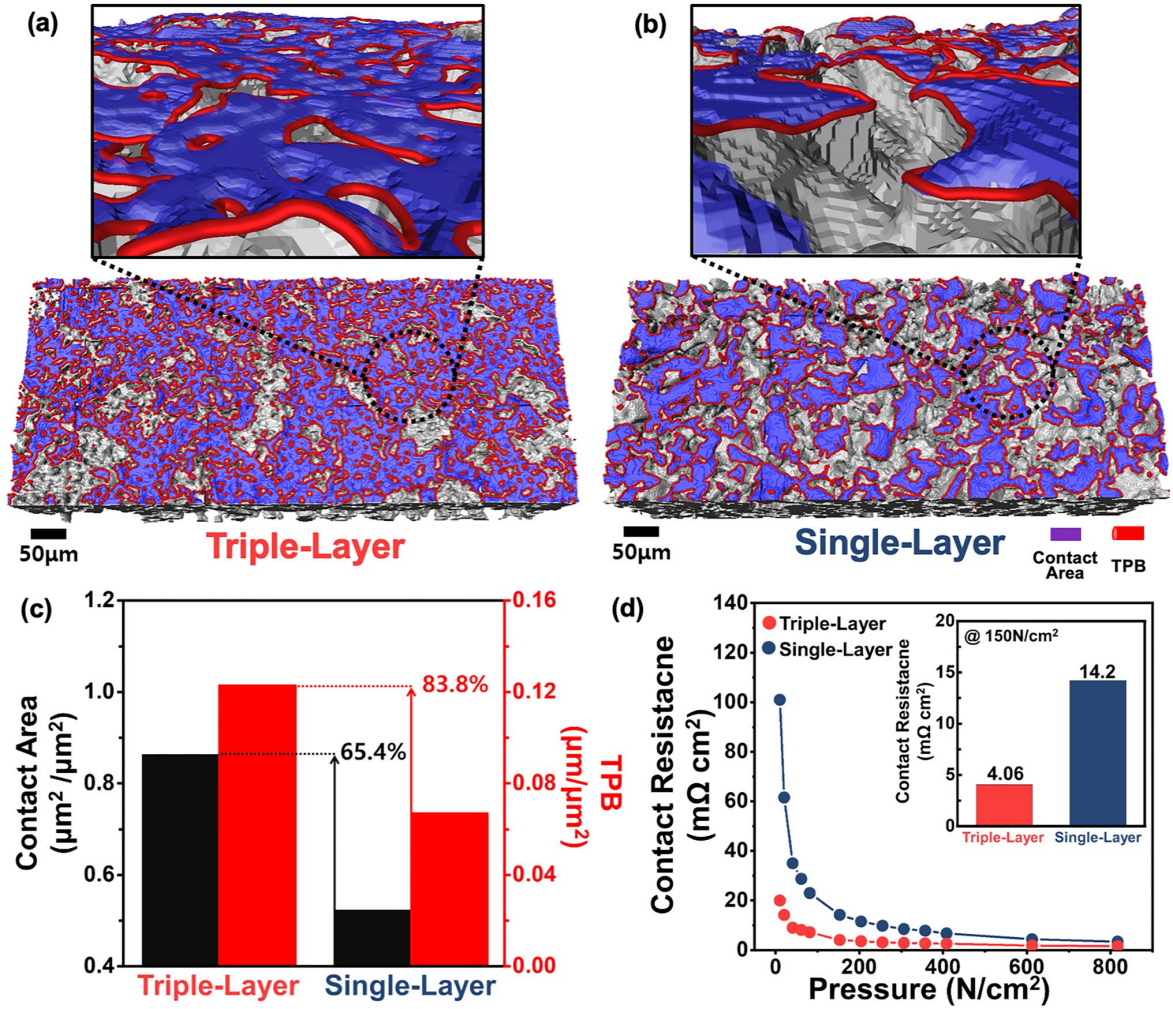
3D reconstructed architecture and detailed view of the interface between the catalyst layer and **a** triple-layer PTL or **b** single-layer PTL in the assembled cell. **c** Quantitative comparison of the contact area and TPB at the interface between the PTL and catalyst layer. **d** Contact resistance with respect to the compression pressure and at the PEMWE cell assembly pressure (150 N cm^−2^) for triple-layer PTL and single-layer PTL

To accurately simulate the interface between the PTL and the catalyst layer, a transparent acrylic cell designed for XRM analysis was prepared, utilizing the same flow field configuration as the PEMWE cell used for electrochemical evaluations (Fig. S7). Figure S8 presents XRM images of the PEMWE components within the assembled transparent cell at different magnifications. These images were used for 3D reconstruction, allowing for a comprehensive analysis of the PTL-catalyst layer interface (Fig. S9). Figure 4a, b visualizes the contact area and the TPB at the contact interface between the catalyst layer and the triple-layer or single-layer PTL, respectively. The TPB represents the region where the catalyst layer, reactants (H_2_O), and Ti-PTL converge, serving as active sites for electrochemical reactions [19, 35, 36]. The MPL surface of the triple-layer PTL demonstrates a larger contact area and a higher number of active sites at the interface with the catalyst layer compared to the single-layer PTL. The detailed views reveal that the smoother MPL surface of the triple-layer PTL establishes a more extensive and highly interconnected contact with the catalyst layer. To further investigate surface characteristics, 3D laser confocal microscopy was employed to analyze the surface of both PTLs (Fig. S10). The results confirmed that the triple-layer PTL’s MPL has lower roughness and more uniform surface properties, contributing to superior interfacial contact with the catalyst layer. This improvement not only minimizes ohmic losses during electrochemical reactions but also prevents membrane damage and deformation caused by the rough PTL surface, ultimately enhancing electrochemical performance. Quantitative analysis reveals that the triple-layer PTL achieved a 65.4% increase in contact area and an 83.8% increase in TPB compared to the single-layer PTL (Fig. 4c). These findings highlight that the MPL pore structure in the triple-layer PTL was tailored to enhance oxygen evolution reaction (OER) kinetics. This aligns with previous study by Peng et al*.* that the MPL pore structure should be optimized for water accessibility, rather than merely increasing contact area with the catalyst layer [7]. Specifically, the numerous micropores in the MPL structure of the triple-layer PTL generate additional TPB regions by facilitating the access of more reactants, thereby increasing active sites for electrochemical reactions. This improvement leads to better utilization of expensive iridium catalysts and reduces kinetic losses. Figure 4d presents the interfacial contact resistance analysis conducted to identify the electrical contact characteristics of the PTL surface. Across the entire range of compression pressures, the triple-layer PTL exhibited lower contact resistance than the single-layer PTL. Notably, at a compression pressure of 150 N cm^−2^, typically for PEMWE cell assembly [29, 33], the contact resistance of the triple-layer PTL was measured at 4.06 mΩ cm^2^, significantly lower than the 14.5 mΩ cm^2^ observed for the single-layer PTL. These results directly demonstrate that the enhanced interfacial properties of the triple-layer PTL effectively reduce ohmic losses during PEMWE operation, thereby improving overall cell performance.

### Mass Transport Behavior Analysis Using Numerical Modeling

**Fig. 5:**
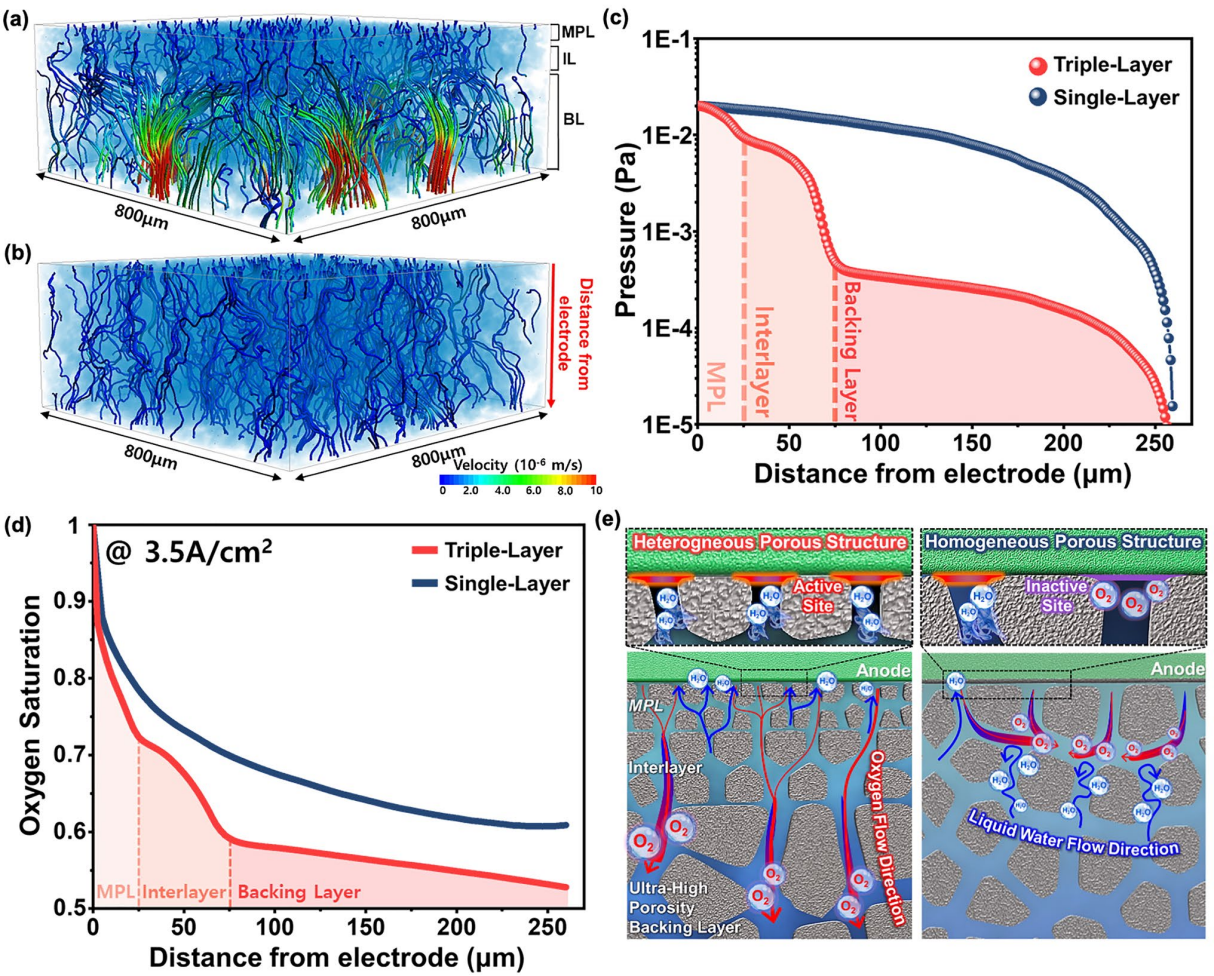
Mass transport characteristics with numerical simulation. Streamlines for the velocity of oxygen transport from the electrode to BPP in the structure of **a** triple-layer PTL and **b** single-layer PTL. **c** Comparison of the pressure of oxygen transport from the electrode to the BPP within the PTL structure as a function of distance from the electrode. **d** Oxygen saturation within the PTL structure as a function of distance from the electrode for oxygen flow at a current density of 3.5 A cm^−2^. **e** Schematics of the oxygen transport behavior in PTLs with heterogeneous and homogeneous porous structures

Figure 5a, b depicts simulated streamlines of oxygen transport velocity from the electrode to the BPP, derived from numerical modeling of digitally twinned 3D PTL structures. The results demonstrate that oxygen transport in the triple-layer PTL occurs at higher velocities along shorter streamline paths compared to the single-layer PTL, indicating more efficient oxygen release. To understand the origins of these differences, oxygen transport pressure along the through-plane direction was analyzed (Fig. 5c). Across the entire triple-layer PTL, the oxygen transport pressure was consistently lower than that in the single-layer PTL, reflecting reduced resistance to oxygen flow and more efficient transport. A steeper pressure drop was observed within the MPL and interlayer of the triple-layer PTL, despite their lower porosity and smaller pore sizes compared to single-layer PTL. This result is attributed to the graded porous structure of the triple-layer PTL. This behavior aligns with the oxygen bubbles’ natural tendency to migrate towards regions with larger pore size and higher porosity, a feature intrinsic to the graded porous structure of the triple-layer PTL. These findings corroborate previous studies emphasizing the role of heterogeneous porous structures, particularly the MPL, in facilitating efficient oxygen release [24, 25, 37, 38]. To isolate the effect of the gradient pore structure, an MPL single-layer PTL with a thickness of 260 µm, matching the MPL pore structure of the triple-layer PTL, was fabricated using a tape casting process. Figure S11 confirms that the MPL single-layer PTL exhibited uniform microstructural properties identical to those of the MPL in the triple-layer PTL. A comparison of oxygen transport pressures between the MPL single-layer PTL and MPL section of the triple-layer PTL under the same conditions revealed that the MPL in the triple-layer PTL experienced lower transport pressures (Fig. S12). This confirms that the graded porous structure, consisting of an interlayer and a backing layer with larger pores and higher porosity, significantly enhances oxygen transport toward the BPP. To further investigate the influence of the ultra-high porosity of the backing layer on oxygen exhaust to the flow channel, additional triple-layer PTLs were fabricated with backing layer porosities of 46% and 56%, while maintaining comparable microstructural properties to the original triple-layer PTL with a 75% backing layer porosity (Figs. S13 and S14). Table S3 confirms that all triple-layer PTLs exhibited similar layer thicknesses, porosity and pore sizes, although variations in the rate of closed pores and tortuosity were observed due to differences in backing layer porosity. A comparative analysis of oxygen transport pressures along the through-plane direction of triple-layer PTLs with different backing layer porosities showed that increasing the porosity of the backing layer led to improved oxygen transport efficiency (Fig. S15). This improvement was influenced by the combined effect of enhanced pore connectivity and reduced tortuosity induced by the higher porosity of the backing layer. Numerical simulations of oxygen saturation distributions within the PTL structures were performed to validate these observations. Figure 5d depicts the oxygen saturation profiles along the thickness direction of the PTLs simulated under the condition of oxygen generation at a high current density of 3.5 A cm^−2^. The triple-layer PTL exhibited consistently lower oxygen saturation across its structure compared to the single-layer PTL. Notably, oxygen saturation near the electrode was significantly reduced, highlighting the effectiveness of the triple-layer PTL in mitigating oxygen accumulation in this critical region. A similar trend was observed among the triple-layer PTLs with varying backing layer porosities, where higher porosity resulted in lower oxygen saturation (Fig. S16). As schematically illustrated in Fig. 5e, these results confirm that the combination of a heterogeneous porous structure and an ultra-high porosity backing layer optimizes oxygen transport throughout the PTL. This efficient oxygen transport mechanism facilitates the replacement of oxygen bubbles with water in active sites, thereby enhancing the OER and improving overall electrochemical performance of the system.

### Electrochemical Characterization for PEM Electrolyzer

**Fig. 6:**
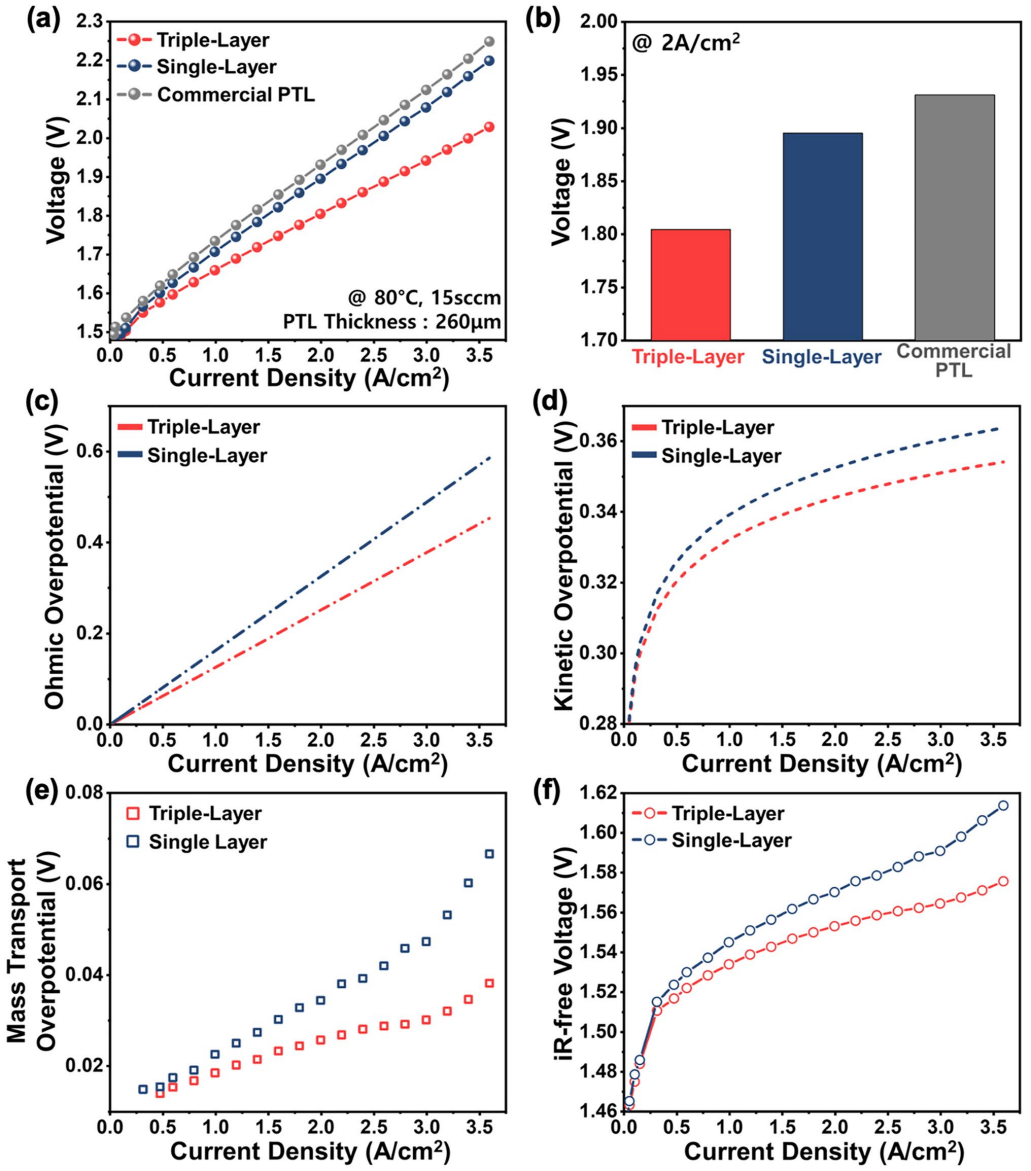
Electrochemical performance evaluation. **a** I-V polarization curves for PEMWE using triple-layer PTL, single-layer PTL, and commercial Ti-PTL. **b** Comparison of cell voltages at a current density of 2 Acm^−2^ for Ti-PTLs. Voltage breakdown analysis of **c** ohmic overpotential, **d** kinetic overpotential, and **e** mass transport overpotential. **f** iR-free polarization curves of triple-layer PTL and single-layer PTL

The electrochemical performance of a PEMWE single cell was evaluated to investigate the impact of the PTL design on cell performance. For comparison, a commercial sintered Ti-powder PTL (Mott Corp., 260 μm thickness), identical in thickness to the triple-layer and single-layer PTLs, was used as a reference. Figure 6a shows the I-V polarization curves of PEMWE cells employing each PTL. The triple-layer PTL consistently outperformed the single-layer PTL and the commercial PTL across the entire range of measured current densities. At a current density of 2 A cm^−2^, the triple-layer PTL exhibited a cell voltage of 1.805 V, which was lower than that of the single-layer PTL (1.895 V) and the commercial PTL (1.931 V) (Fig. 6b). Furthermore, the triple-layer PTL exhibited comparably excellent performance relative to previously reported multilayer PTLs (Fig. S17). This enhanced performance can be attributed to the improved interfacial properties between the triple-layer PTL and the catalyst layer, as well as superior mass transport characteristics. The triple-layer PTL achieved a 127 mV reduction in cell voltage compared to the commercial PTL. Furthermore, from the perspective of operating expenditures (OPEX), an 8.57% improvement in electrical efficiency was observed at a current density of 3 A cm^−2^ (Table S4) [39]. To better understand the impact of PTL microstructural properties on the electrochemical reactions in PEMWE cells, a voltage breakdown analysis was conducted. Figure S18 presents the Tafel plots, providing insight into the breakdown of overpotentials. Figure 6c demonstrates that the triple-layer PTL exhibited lower ohmic overpotential than the single-layer PTL. This reduction is attributed to its improved contact with the catalyst layer provided by the MPL structure in the triple-layer PTL. The low interfacial contact resistance, as shown in Fig. 4d, contributed to minimizing ohmic losses during electrochemical reactions. In terms of kinetic overpotential, Fig. 6d shows that the triple-layer PTL demonstrates lower kinetic overpotential over the entire range of current densities. This improvement highlights the enhanced catalyst utilization enabled by the triple-layer PTL’s design. Furthermore, the mass transport overpotential, illustrated in Fig. 6e, reveals the superior mass transport properties of the triple-layer PTL. At high current densities, the gradient pore structure and ultra-high porosity backing layer of the triple-layer PTL facilitated oxygen bubble release toward the BPP, significantly reducing mass transport losses. Figure 6f displays the iR-free voltage across the entire range of current densities. The triple-layer PTL’s combined features-such as an increased TPB due to its MPL design and the prevention of oxygen bubble accumulation at the catalyst layer interface, enabled by its optimized pore structure-resulted in the generation of additional active sites for electrochemical reactions. In addition, long-term durability testing conducted at a constant current density of 2 A cm^−2^ for 350 h confirmed the excellent stability of the triple-layer PTL, which exhibited a degradation rate of 15.1 μV h^−1^ (Fig. S19). Figure S20 presents the cross-sectional SEM image of the triple-layer PTL after the long-term test, revealing that the layered structure remained without any noticeable deformation or collapse, thereby demonstrating its high mechanical integrity. Furthermore, the XRD pattern of the triple-layer PTL after the long-term stability test, as shown in Fig. S21, revealed only peaks corresponding to phase-pure Ti and Pt from the protective coating used to prevent the formation of an oxide layer, with no additional peaks observed, thereby confirming its excellent chemical stability. To further assess the effect of backing layer porosity on electrochemical performance, triple-layer PTLs with varying backing layer porosities (46%, 56%, and 75%) were evaluated. As shown in Fig. S22, the performance of the triple-layer PTLs improved at all current densities as the porosity of the backing layer increased. This improvement is attributed to enhanced mass transport efficiency provided by the ultra-high porosity backing layer, which increased the availability of active sites for electrochemical reactions. The results highlight the indispensability of designing an interlayer to achieve an ultra-high porosity microstructure in the backing layer, enabling efficient water electrolysis.

## Conclusion

In this study, we successfully developed a novel triple-layer Ti-PTL with a pore-graded structure and an ultra-high porosity backing layer for the first time, utilizing a practical tape casting process combined with a roll calendering procedure. This manufacturing approach is highly feasible for commercial PTL fabrication. Advanced 3D reconstruction technology using XRM enabled the digital twinning of the interfacial morphology formed between the catalyst layer and PTL within an assembled PEMWE cell. This analysis provided direct insights into the MPL’s strategic design, which enhances catalyst utilization by creating a uniform, low-roughness surface that increases the contact area and facilitates the formation of more TPBs through its microporous structure. Numerical simulations further confirmed that the graded porous structure and ultra-high porosity backing layer of the triple-layer PTL significantly promote oxygen release, ensuring efficient mass transport and expanding the availability of active sites for electrochemical reactions. Electrochemical performance evaluations of the PEMWE cell demonstrated that the novel triple-layer PTL effectively reduced ohmic, kinetic, and mass transport overpotentials, resulting in superior overall cell performance. The high-performance triple-layer PTL, with the cost-effective and viable manufacturing process, offers a breakthrough in accelerating the commercialization of PEMWE technology and supporting the global transition to sustainable energy systems.

## Supplementary Information

Below is the link to the electronic supplementary material.

Supplementary file1 (DOCX 8169 KB)
