# Supplementary material for: Triple-Layer Porous Transport Layers with Ultra-High Porosity for Enhanced Oxygen Transport and Catalyst Utilization in Water Electrolysis
Source: Nanomicro Lett. 2025 Jun 30;17:316. doi: 10.1007/s40820-025-01831-z (PMC12209109; doi:10.1007/s40820-025-01831-z)
Supplement: Supplementary file 1 — Supplementary file1 (DOCX 8169 KB) [file 40820_2025_1831_MOESM1_ESM.docx]

Supporting Information for

**Triple-Layer Porous Transport Layers with Ultra-High Porosity for Enhanced Oxygen Transport and Catalyst Utilization in Water Electrolysis**

Seong Hyun Park^1†^, Young Je Park^1,2†^, Seungsoo Jang^1^, Pilyoung Lee^3^, Soobin Yoon^3^, Young-June Park^3,^*, Chi-Young Jung^2,^*, and Kang Taek Lee^1,4,^*

^1^Department of Mechanical Engineering, KAIST, Daejeon 34141, Republic of Korea

^2^Hydrogen Research and Demonstration Center, Hydrogen Energy Institute, Korea Institute of Energy Research (KIER), Jeollabuk-do 56332, Republic of Korea

^3^Hydrogen and Fuel Cell Development Center, Hyundai Motor Group, Gyeonggi-do 16891, Republic of Korea

^4^KAIST Graduate School of Green Growth & Sustainability, Daejeon 34141, Republic of Korea

^†^Seong Hyun Park and Young Je Park contributed equally to this work.

*Corresponding authors. E-mail: [yjpark2935@hyundai.com](mailto:yjpark2935@hyundai.com) (Young-June Park); [cyjung@kier.re.kr](mailto:cyjung@kier.re.kr) (Chi-Young Jung); [leekt@kaist.ac.kr](mailto:leekt@kaist.ac.kr) (Kang Taek Lee)

**Supplementary Tables and Figures**

**Table S1** Summary of the quantitative microstructural properties of triple-layer PTL and single-layer PTL


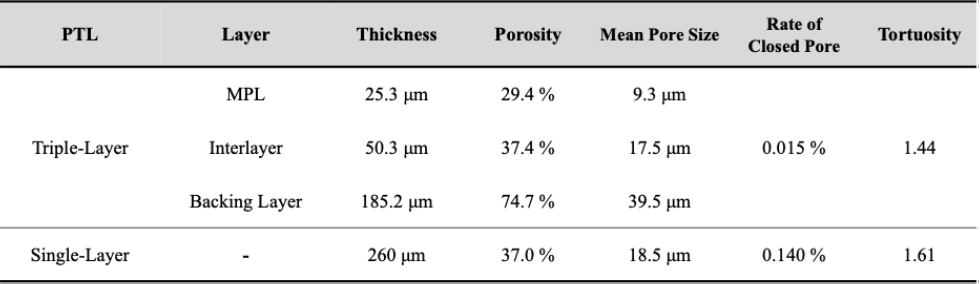


**Table S2** Comparison of maximum porosity of sintered powder PTLs [S1-S7]

**Table S3** Summary of the quantitative microstructural properties of triple-layer PTLs with different porosities of backing layers

**Table S4** Comparison of electrical efficiency at a current density of 3 A/cm²

**Fig. S1** TGA curve of triple-layer tape at 400 °C ~ 800 °C

**Fig. S2** Surface SEM images of (**a**) MPL side and (**b**) ultra-high porosity backing layer side of a triple-layer PTL

**Fig. S3** SEM images of (**a**) surface and (**b**) cross-section of the single-layer PTL


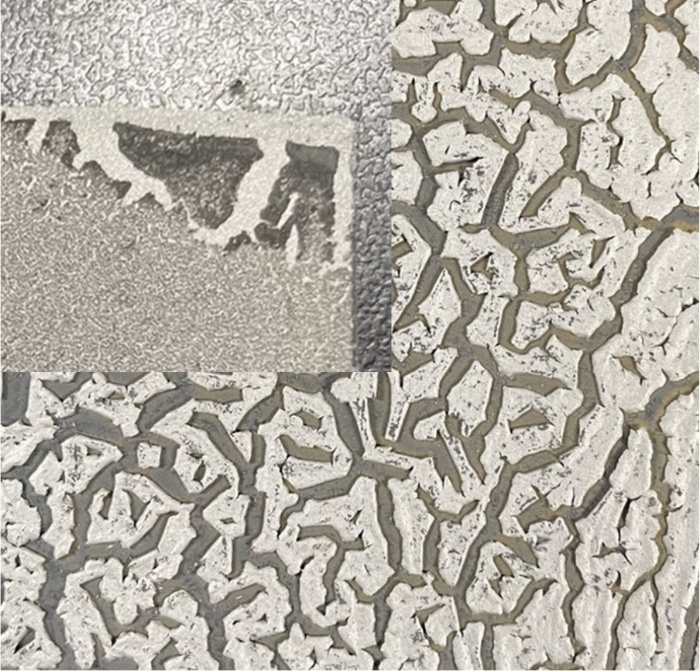


**Fig. S4** Photographs of a dual-layer PTL with an ultra-high porosity backing layer after heat treatment process, and delamination between MPL and backing layer

**Fig. S5** Compressive stress–strain curve of the triple-layer


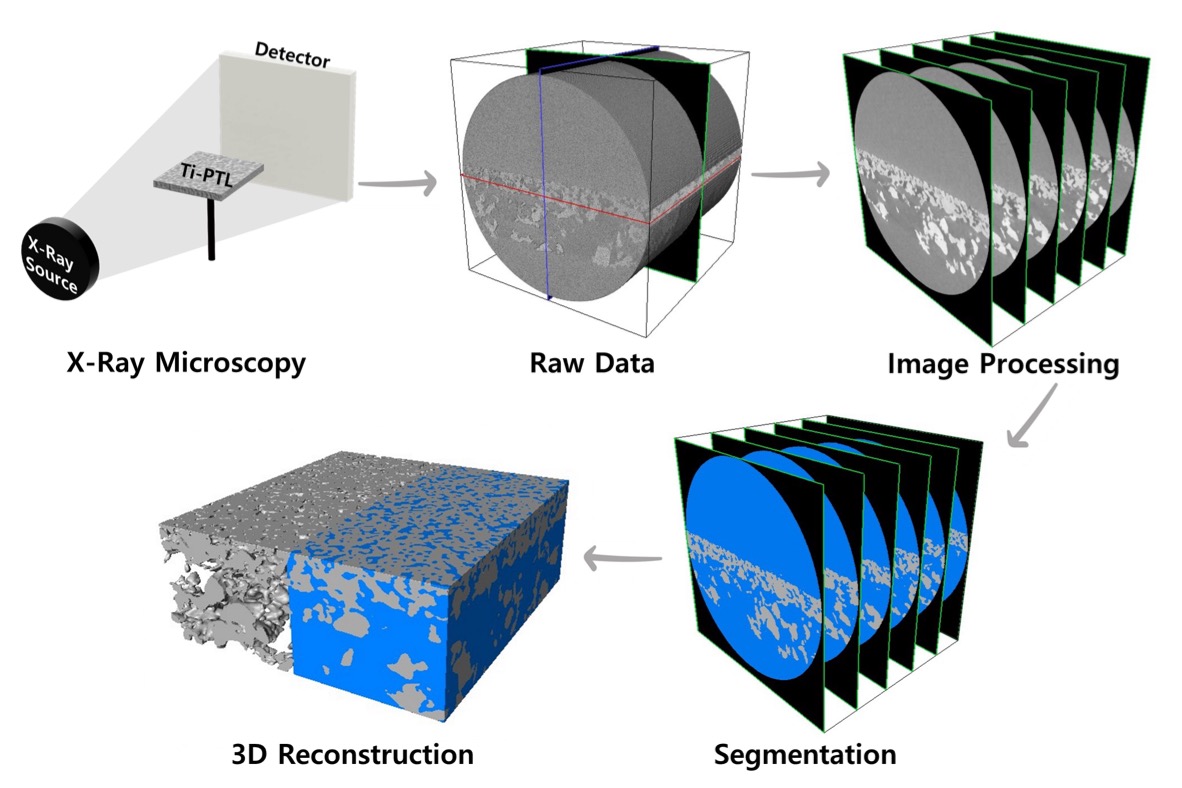


**Fig. S6** Workflow of the digital twinning process for 3D reconstruction using XRM


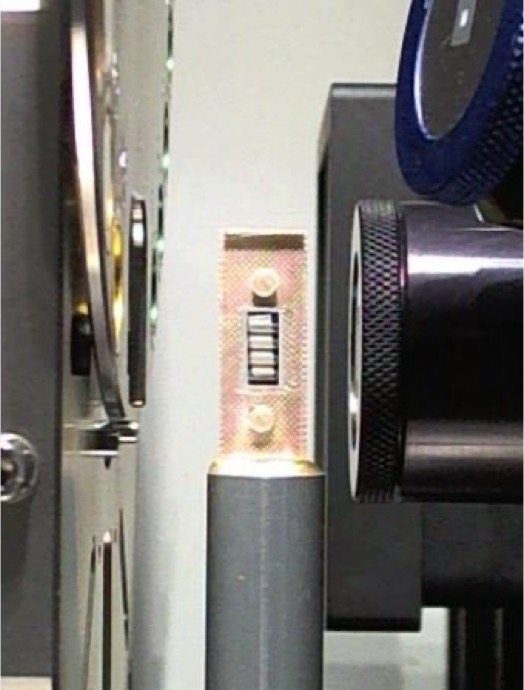


**Fig. S7** Transparent acrylic cell for XRM analysis


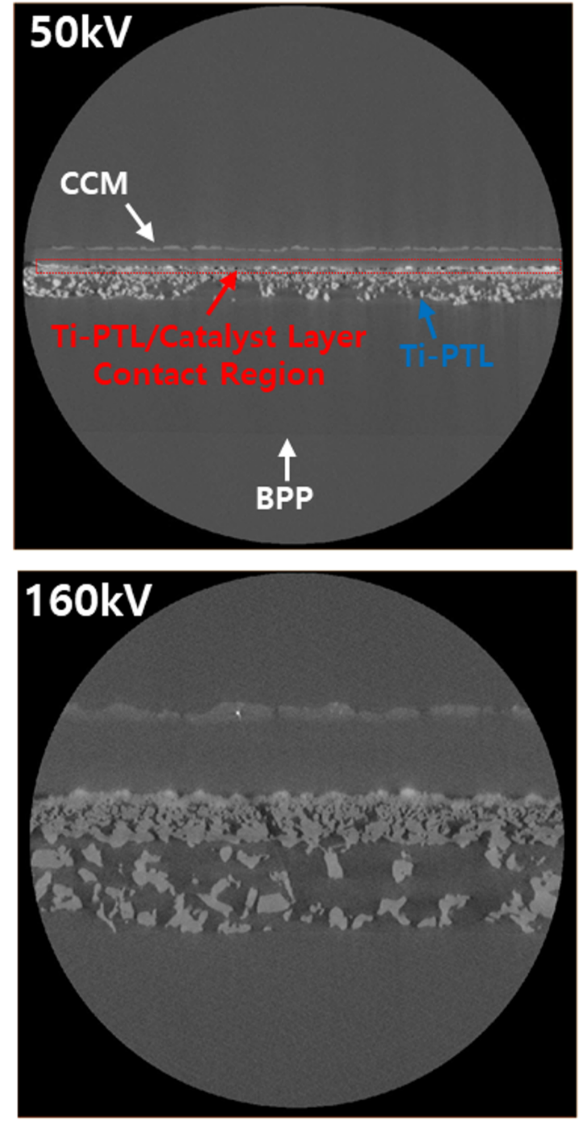


**Fig. S8** XRM images of PEMWE components within the assembled cell

**
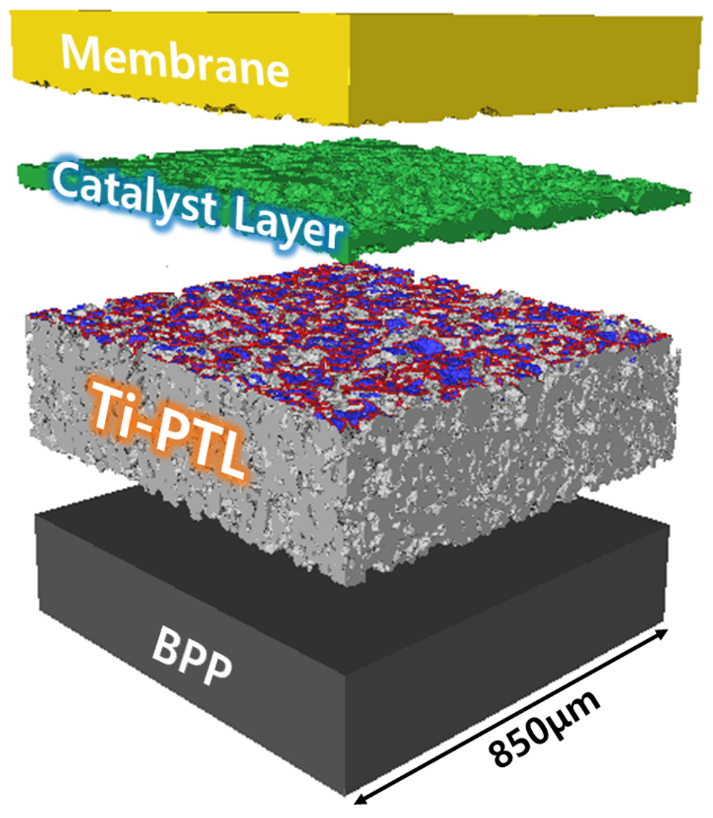
**

**Fig. S9** 3D reconstructed architectures of the membrane, catalyst layer, Ti-PTL, and BPP


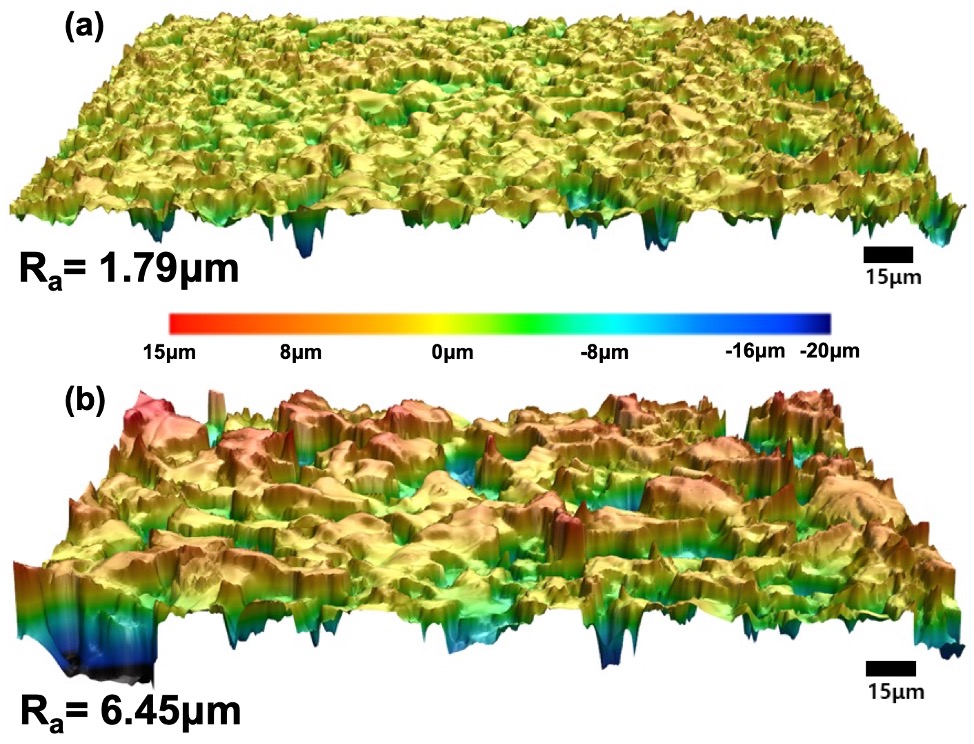


**Fig. S10** 3D laser confocal microscope images and quantified roughness values of (**a**) the MPL surface in the triple-layer PTL and (**b**) the surface of the single-layer PTL

**
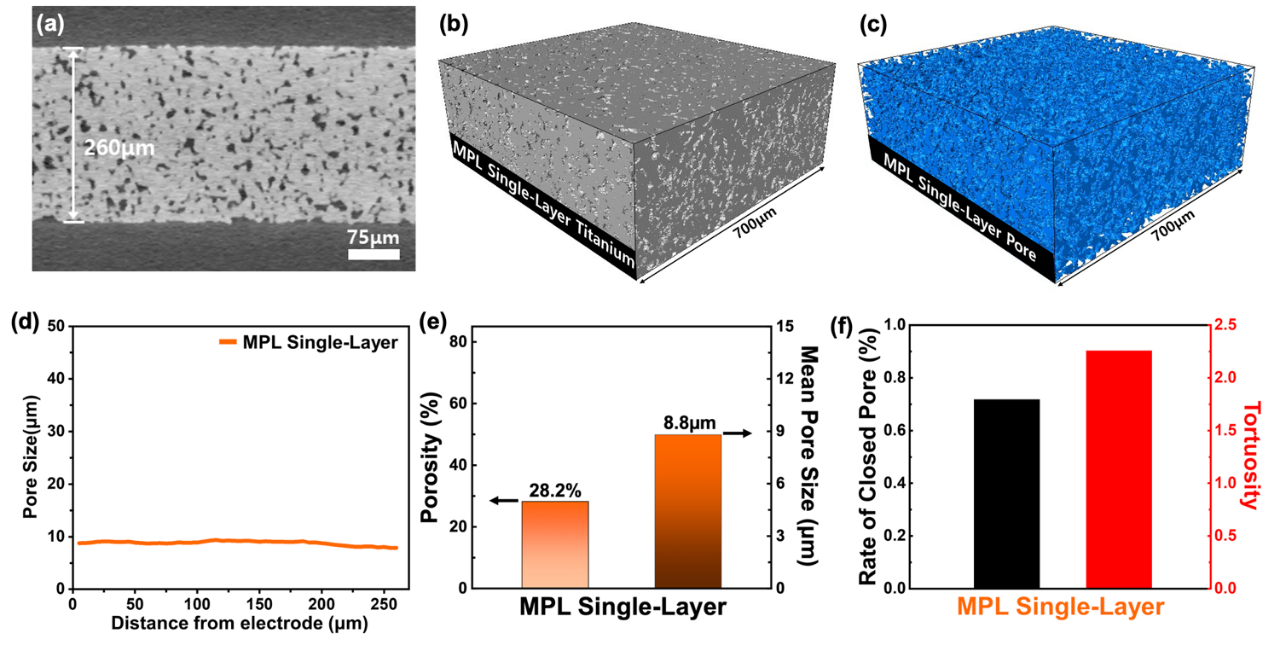
**

**Fig. S11** Pore-structural characteristics of the MPL single-layer PTL (260 µm). (**a**) Cross-sectional SEM image of the MPL single-layer PTL. 3D reconstructions of (**b**) titanium phase and (**c**) pore phase in MPL single-layer PTL. (**d**) Local pore size as a function of distance from the electrode. (**e**) Porosity and mean pore size of the MPL single-layer PTL. (**f**) Rate of closed pores and tortuosity of the MPL single-layer PTL


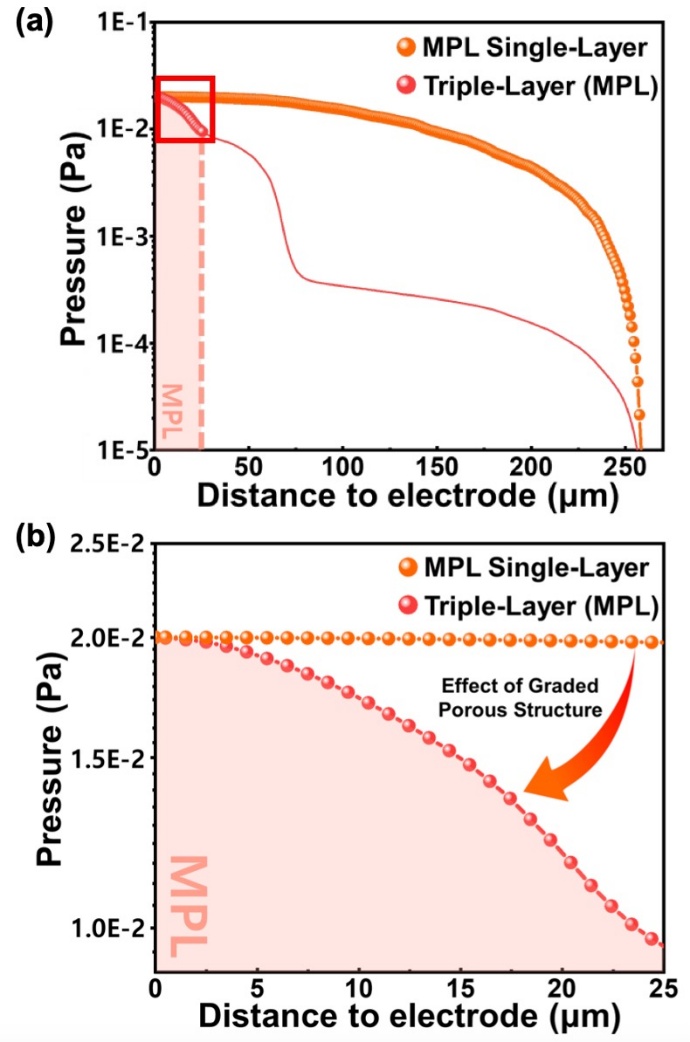


**Fig. S12** (**a**) Comparison of the pressure of oxygen transport between MPL single-layer PTL and MPL in triple-layer PTL. (**b**) A zoomed-in view of the pressure of oxygen transport at the MPL region (~25 μm)

**
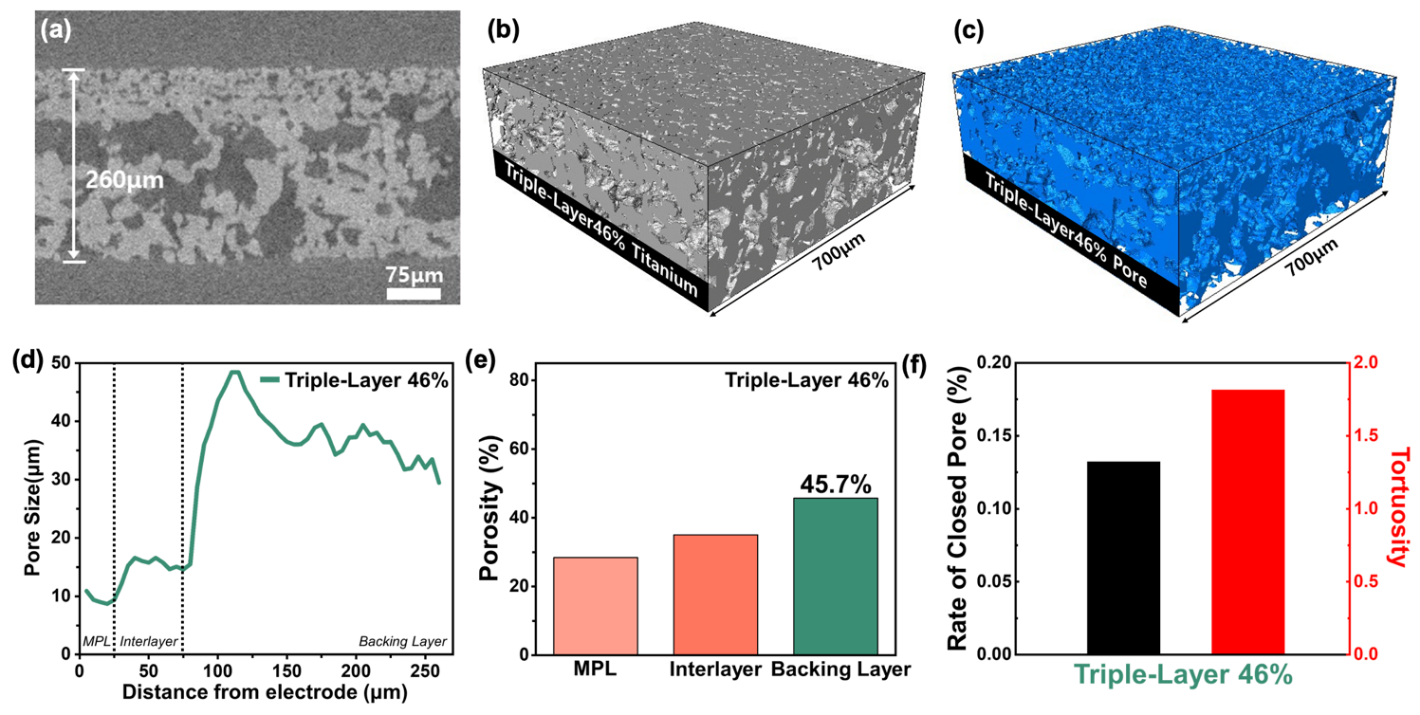
**

**Fig. S13** Pore-structural characteristics of the triple-layer 46% PTL. (**a**) Cross-sectional SEM image of the triple-layer 46% PTL. 3D reconstructions of (**b**) titanium phase and (**c**) pore phase in triple-layer 46% PTL. (**d**) Local pore size as a function of distance from the electrode. (**e**) Porosity by layer within the triple-layer 46% PTL. (**f**) Rate of closed pores and tortuosity of the triple-layer 46% PTL

**
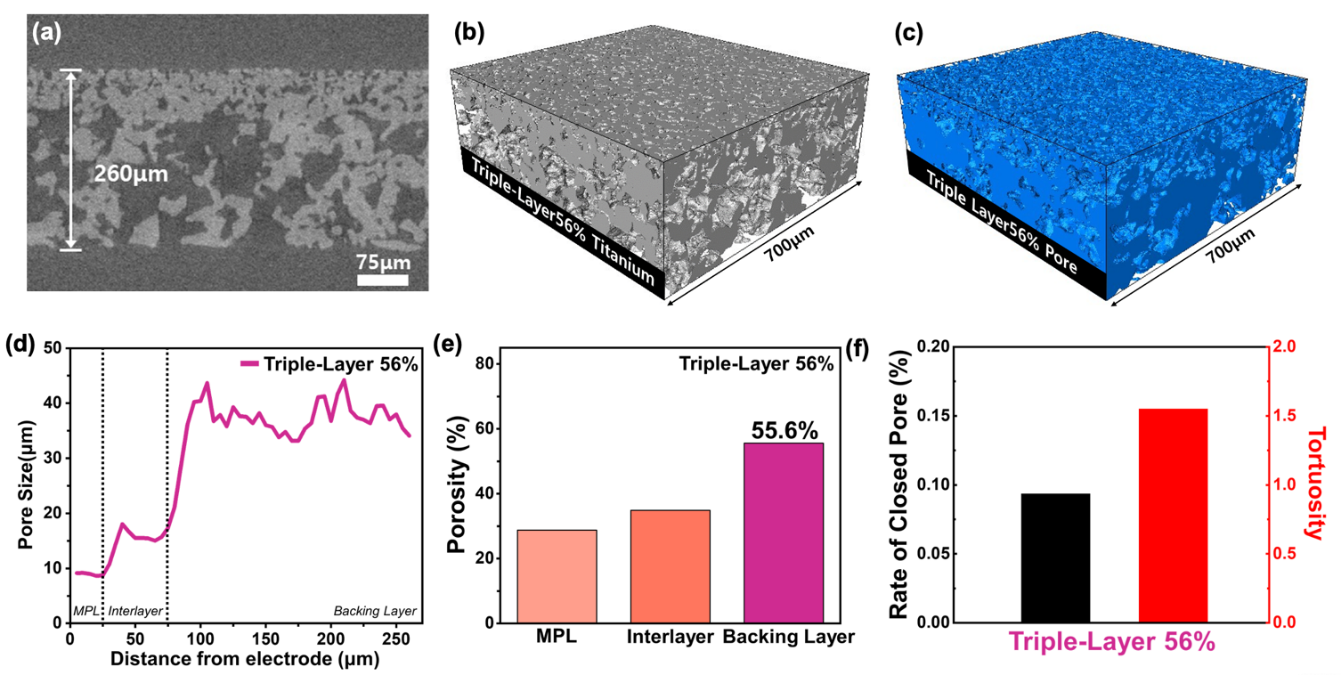
**

**Fig. S14** Pore-structural characteristics of the triple-layer 56% PTL. (**a**) Cross-sectional SEM image of the triple-layer 56% PTL. 3D reconstructions of (**b**) titanium phase and (**c**) pore phase in triple-layer 56% PTL. (**d**) Local pore size as a function of distance from the electrode. (**e**) Porosity by layer within the triple-layer 56% PTL. (**f**) Rate of closed pores and tortuosity of the triple-layer 56% PTL.

**
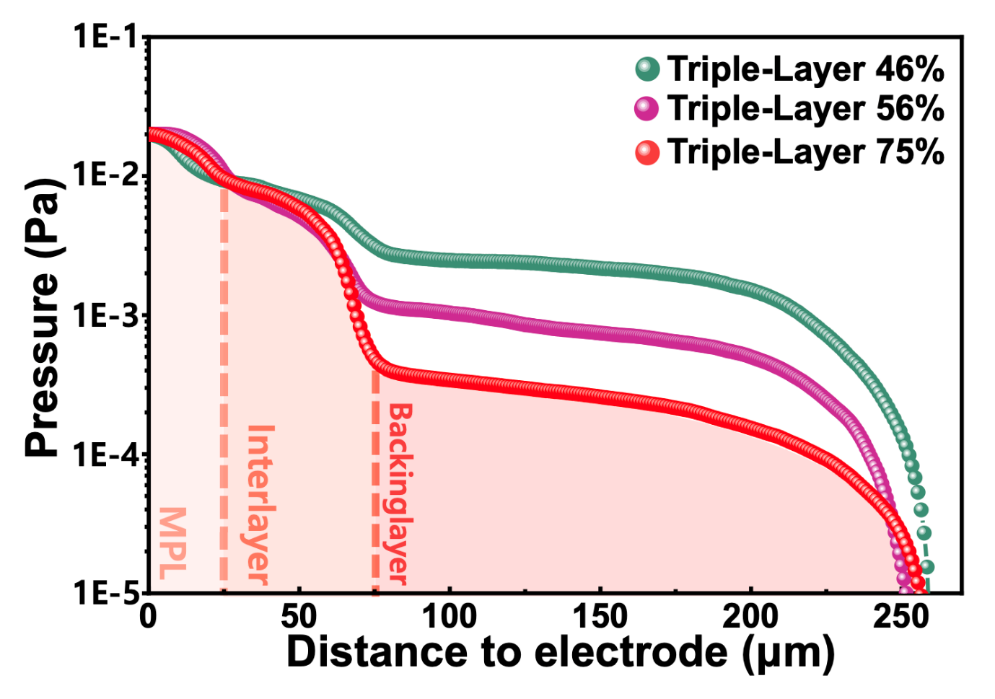
**

**Fig. S15** Comparison of oxygen transport pressure in triple-layer PTLs with different backing layer porosities (46%, 56%, and 75%)


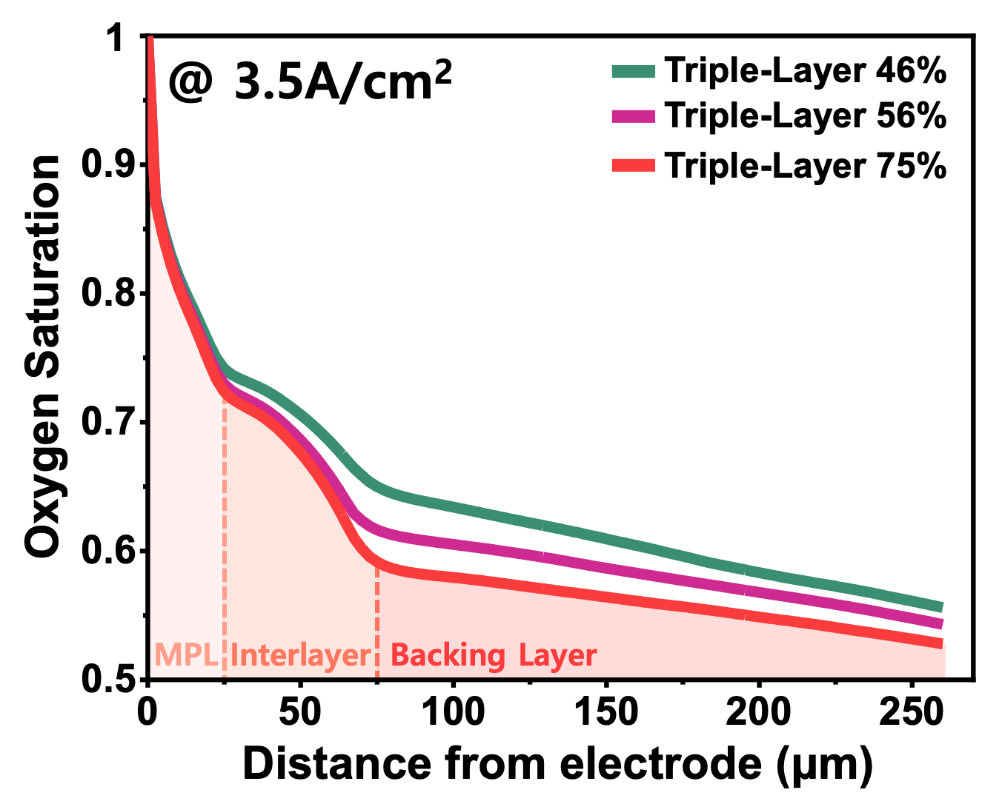


**Fig. S16** Comparison of oxygen saturation in triple-layer PTLs with different backing layer porosities (46%, 56%, and 75%) at a current density of 3.5 A/cm²

**Fig. S17** Comparison of cell voltages at 2 A/cm² with previous studies on multilayer PTLs [S7-S13]

c

**Fig. S18** Tafel plots of iR-free voltage at low current densities using triple-layer PTL and single-layer PTL

**Fig. S19** Long-term stability test of the triple-layer PTL at a current density of 2 A/cm²


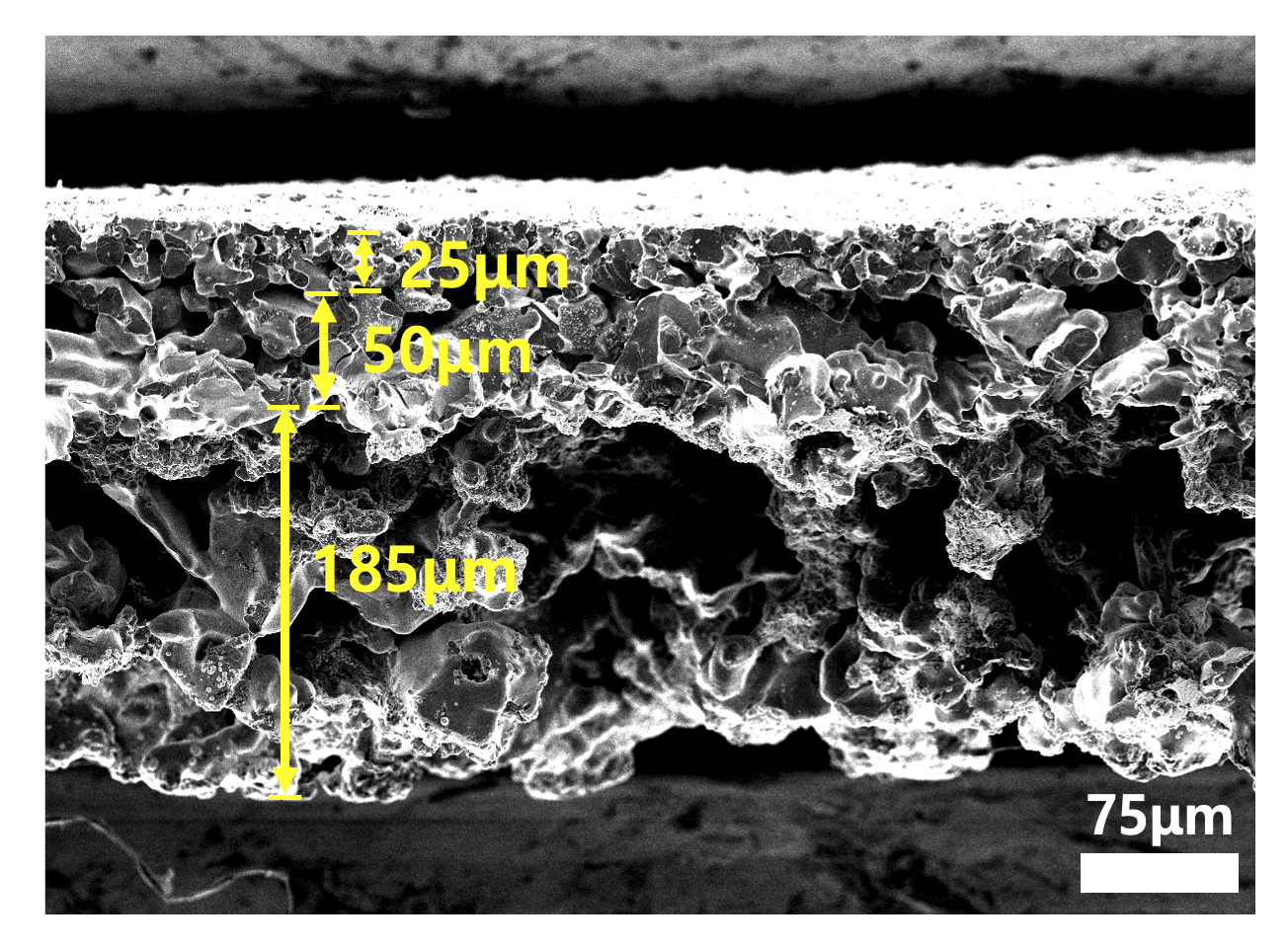


**Fig. S20** Cross-sectional SEM image of the triple-layer PTL after the long-term stability test

**Fig. S21** XRD pattern of the triple-layer PTL after the long-term stability test

**Fig. S22** I-V polarization curves for PEM water electrolysis using triple-layer PTLs with different backing layer porosities (46, 56, and 75%)

**Supplementary References**

1. B. Zhang, L. Shen, X. Duan, X. Liu, J. Wang, Large-scale ultra-thin titanium porous transport layers fabricated by powder rolling for polymer electrolyte membrane water electrolysis. Int. J. Hydrog. Energy **116**, 64–72 (2025). <https://doi.org/10.1016/j.ijhydene.2025.02.459>
2. F.J. Hackemüller, E. Borgardt, O. Panchenko, M. Müller, M. Bram, Manufacturing of large-scale titanium-based porous transport layers for polymer electrolyte membrane electrolysis by tape casting. Adv. Eng. Mater. **21**(6), 1801201 (2019). <https://doi.org/10.1002/adem.201801201>
3. S.A. Grigoriev, P. Millet, S.A. Volobuev, V.N. Fateev, Optimization of porous current collectors for PEM water electrolysers. Int. J. Hydrog. Energy **34**(11), 4968–4973 (2009). <https://doi.org/10.1016/j.ijhydene.2008.11.056>
4. M. Fakourihassanabadi, B. Guerreiro, J. Gaudet, M.H. Martin, S. Abbasi et al., Fabrication of a Ti-based 3D porous transport layer for PEMWEs using ShockWave-induced spraying and cold spray. Surf. Coat. Technol. **477**, 130353 (2024). <https://doi.org/10.1016/j.surfcoat.2023.130353>
5. J.K. Lee, G.Y. Lau, M. Sabharwal, A.Z. Weber, X. Peng et al., Titanium porous-transport layers for PEM water electrolysis prepared by tape casting. J. Power Sources **559**, 232606 (2023). <https://doi.org/10.1016/j.jpowsour.2022.232606>
6. K. Bobzin, S. Finger, L. Zhao, H. Heinemann, E. Olesch et al., Porosity-zoned porous-transport layer for proton-exchange membrane water electrolysis by high-velocity flame spraying. Adv. Eng. Mater. **27**(5), 2402462 (2025). <https://doi.org/10.1002/adem.202402462>
7. P. Lettenmeier, S. Kolb, N. Sata, A. Fallisch, L. Zielke et al., Comprehensive investigation of novel pore-graded gas diffusion layers for high-performance and cost-effective proton exchange membrane electrolyzers. Energy Environ. Sci. **10**(12), 2521–2533 (2017). <https://doi.org/10.1039/c7ee01240c>
8. P. Lettenmeier, S. Kolb, F. Burggraf, A.S. Gago, K.A. Friedrich, Towards developing a backing layer for proton exchange membrane electrolyzers. J. Power Sources **311**, 153–158 (2016). <https://doi.org/10.1016/j.jpowsour.2016.01.100>
9. S. Stiber, H. Balzer, A. Wierhake, F.J. Wirkert, J. Roth et al., Porous transport layers for proton exchange membrane electrolysis under extreme conditions of current density, temperature, and pressure. Adv. Energy Mater. **11**(33), 2100630 (2021). <https://doi.org/10.1002/aenm.202100630>
10. C.C. Weber, S. De Angelis, R. Meinert, C. Appel, M. Holler et al., Microporous transport layers facilitating low iridium loadings in polymer electrolyte water electrolysis. EES Catal. **2**(2), 585–602 (2024). <https://doi.org/10.1039/d3ey00279a>
11. T. Schuler, J.M. Ciccone, B. Krentscher, F. Marone, C. Peter et al., Hierarchically structured porous transport layers for polymer electrolyte water electrolysis. Adv. Energy Mater. **10**(2), 1903216 (2020). <https://doi.org/10.1002/aenm.201903216>
12. T. Deng, H. Huang, L. Fan, S. Xu, H. Li, Porous transport layers with TiC-coated microporous layers for proton exchange membrane water electrolysis. ACS Sustainable Chem. Eng. **11**(48), 17075–17085 (2023). <https://doi.org/10.1021/acssuschemeng.3c05256>
13. Y. Tang, S. Su, X. Niu, Z. Song, W. Li, A gradient porous transport layer enabling a high-performance proton-exchange membrane electrolysis cell. Renew. Energy **237**, 121707 (2024). <https://doi.org/10.1016/j.renene.2024.121707>
